# An interpretable machine learning model for predicting sepsis risk in ICU patients with non-traumatic subarachnoid hemorrhage: development and validation using the MIMIC-IV database

**DOI:** 10.3389/fneur.2026.1734264

**Published:** 2026-01-21

**Authors:** Shaojie Guo, Yang Liu, Jing Xia, Ang Li, Xinchen Ma, Yong Chen, Jv Wang, Bingsha Han, Gaofeng Li, Guang Feng

**Affiliations:** 1 Graduate School of Xinxiang Medical University, Xinxiang, China; 2 Department of Neurosurgical Intensive Care Unit, Henan Provincial People’s Hospital, Zhengzhou, China

**Keywords:** machine learning, MIMIC-IV database, prediction model, sepsis, subarachnoid hemorrhage

## Abstract

**Objective:**

This study aimed to develop and validate a machine learning (ML) prediction model for assessing the risk of sepsis in intensive care unit (ICU) patients with non-traumatic subarachnoid hemorrhage (SAH), thereby providing a reference for the early clinical identification of high risk patients.

**Methods:**

We conducted a retrospective cohort study using data from the Medical Information Mart for Intensive Care (MIMIC-IV) database, which includes admissions between 2008 and 2022. We extracted demographic information, laboratory parameters, complications, and other clinical data. Patients were randomly divided into a training set and a test set in an 8:2 ratio. Least Absolute Shrinkage and Selection Operator regression was used to identify core predictive features. Fourteen machine learning models were constructed, including Random Forest, Gradient Boosting, Kernel-based SVM, Logistic Regression, K-Nearest Neighbors, Partial Least Squares, Boosting Method, Neural Network, Naive Bayes, Discriminant Analysis, Lasso, XGBoost, CATBoost, and LightGBM. Key evaluation metrics included sensitivity, specificity, accuracy, F1 score, Youden index, and the area under the curve (AUC). SHapley Additive exPlanations (SHAP) analysis was employed to interpret the model’s decision logic, and Decision Curve Analysis (DCA) was used to assess clinical utility.

**Results:**

A total of 1,052 patients with non-traumatic SAH were enrolled, with 841 assigned to the training set and 211 to the test set. Lasso regression identified 11 core predictive features, including pneumonia, norepinephrine use, mechanical ventilation, Glasgow Coma Scale (GCS) grade, and acute kidney injury (AKI). The CATBoost model demonstrated the best performance: in the training set, it achieved an AUC of 88.9%, sensitivity of 73.2%, specificity of 85.9%, and a Youden index of 0.592; in the test set, it achieved an AUC of 0.887, sensitivity of 75.5%, specificity of 82.3%, and a Youden index of 0.578. Performance fluctuation between the training and test sets was less than 2%, indicating excellent stability. SHAP analysis revealed that pneumonia, norepinephrine use, and mechanical ventilation were the top three features influencing sepsis risk, with pneumonia significantly increasing the risk. DCA results showed that the CATBoost model had the highest net benefit in the high-risk threshold range of 0.2–0.6.

**Conclusion:**

The machine learning model developed based on the MIMIC-IV database can effectively predict the risk of sepsis in ICU patients with non-traumatic SAH. It demonstrates good interpretability and clinical utility, providing a basis for clinical risk stratification and precise intervention.

## 1 Introduction

Subarachnoid hemorrhage (SAH), particularly non-traumatic SAH, is a severe neurological emergency characterized by acute bleeding into the subarachnoid space, most commonly caused by the rupture of an intracranial aneurysm (1). This condition is associated with high mortality, and survivors often experience severe long-term disability, including permanent neurological deficits, cognitive impairment, and a significantly reduced quality of life (2). The pathophysiology of SAH is complex, involving not only the direct effects of hemorrhage but also a series of secondary injuries that critically influence patient prognosis.

Among the serious complications affecting SAH patients, sepsis is particularly prominent. Sepsis is a life-threatening systemic inflammatory response triggered by infection and is highly prevalent in critically ill patients (3). It can exacerbate the already compromised physiological state of SAH patients, leading to multiple organ dysfunction and further increasing mortality risk (4). The relationship between SAH and sepsis is complex, involving multiple pathophysiological mechanisms such as immune response dysregulation, autonomic dysfunction, and increased susceptibility to infection due to prolonged hospitalization and invasive procedures (2, 3).

Although sepsis is a major complication of SAH with significant clinical implications, the literature notably lacks dedicated sepsis risk prediction models for this specific patient population. Previous studies have identified sepsis as an independent risk factor for mortality in patients with non-traumatic SAH (3). A recent study developed a nomogram for SAH patients with an AUC of 0.824–0.854 (5) but lacked interpretability and comparison with multiple machine learning algorithms. However, no study has comprehensively evaluated the utility of machine learning (ML) methods for predicting sepsis risk in this vulnerable group.

In summary, developing accurate sepsis risk prediction models for patients with non-traumatic SAH is of great clinical importance. Early identification of high-risk individuals facilitates targeted monitoring, timely intervention, and improved patient outcomes through preventive measures. Furthermore, Our SAH-specific sepsis prediction model optimizes ICU resource allocation by identifying high-risk patients, such as those with pneumonia, mechanical ventilation, or low GCS scores, who can then receive intensified monitoring and targeted interventions including frequent vital-sign checks, serial inflammatory markers, early antibiotic stewardship, and ventilator bundles. For low-risk patients, the model helps reduce unnecessary interventions, such as broad-spectrum antibiotic overuse, thereby reallocating resources to those most in need.

## 2 Methods

### 2.1 Data source

The source of data for this study was MIMIC-IV (v3.0), a large-scale, open-source database developed and maintained by the MIT Computational Physiology Laboratory (Johnson, A., Bulgarelli, L., Pollard, T., Gow, B., Moody, B., Horng, S., Celi, L. A., & Mark, R., 2024. MIMIC-IV (version 3.0). PhysioNet. https://doi.org/10.13026/hxp0-hg59). This database contains comprehensive clinical records of all patients admitted to Beth Israel Deaconess Medical Center (Boston, MA) between 2008 and 2022, including laboratory results, vital signs, medication administration, and other relevant clinical data. All personal identifiers were replaced with randomized codes to protect patient confidentiality, ensuring the data were fully deidentified. Thus, neither ethical approval nor patient consent was required for this study.

Access to the MIMIC-IV (v3.0) database was obtained via the official PhysioNet platform. One of the authors, Gaofeng Li, successfully completed the required Collaborative Institutional Training Initiative (CITI) courses (including “Conflict of Interest” and “Data or Sample Only Research”) and obtained authorization to access and use the database (CITI Program ID: 54026276). This study was conducted in compliance with the principles of the Declaration of Helsinki and followed the Strengthening the Reporting of Observational Studies in Epidemiology (STROBE) guidelines.

**Figure 1:**
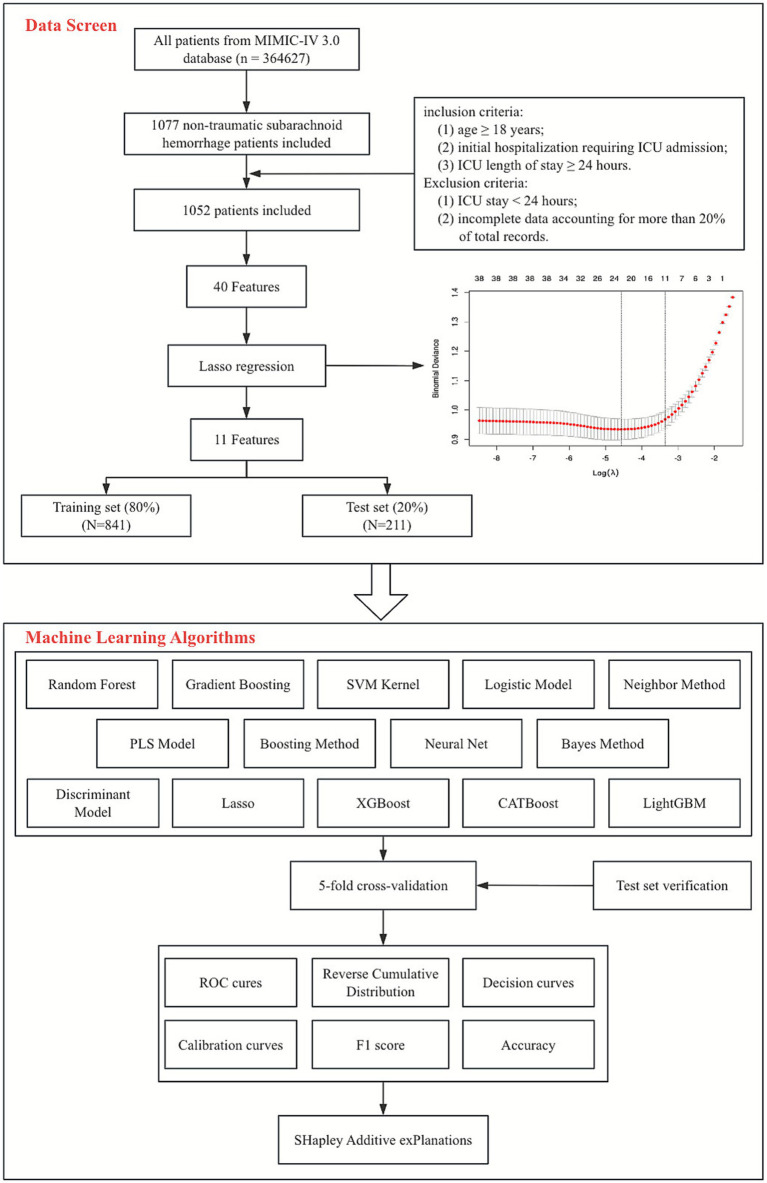
The overall flowchart of the study.

From the database, 1,077 patients with non-traumatic SAH were initially identified using ICD-9 code 430, and ICD-10 codes I602, I604, I606, I607, I608, I609, I6001, I6002, I6010, I6011, I6012, I6020, I6021, I6022, I6031, I6032, I6051, I6052, I6900, I6901, I69011, I69018, I69020, I69021, I69022, I69028, I69044, I69051, I69054, I69092, and I69098. The study population included patients who met the following inclusion criteria: age ≥18 years; initial hospitalization requiring ICU admission; ICU length of stay ≥24 h. Exclusion criteria were: ICU stay <24 h; incomplete data accounting for more than 20% of total records. After applying these criteria, 1,052 patients were enrolled in the study, and the sample selection process is detailed in Figure 1.

### 2.2 Data extraction

All relevant variables were extracted from the MIMIC-IV database using structured query language (SQL) in the PostgreSQL system. Data collected within the first 24 h of ICU admission included demographic variables including age, sex, and race, vital signs including systolic blood pressure (SBP), diastolic blood pressure (DBP), mean arterial pressure (MAP), temperature, heart rate (HR), respiratory rate (RR), and pulse oxygen saturation (SpO_2_), comorbidities including hypertension, diabetes, myocardial infarction, malignancy, chronic kidney disease, and pneumonia, mechanical ventilation use, medication use including dopamine, epinephrine, and norepinephrine, laboratory indicators including white blood cell count (WBC), red blood cell count (RBC), platelet count, hemoglobin, red blood cell distribution width (RDW), sodium, potassium, magnesium, calcium, chloride, glucose, anion gap (AG), prothrombin time (PT), activated partial thromboplastin time (APTT), International Normalized Ratio (INR), blood urea nitrogen (BUN), and creatinine, and scoring systems including Glasgow Coma Scale (GCS) grade and Charlson Comorbidity (used to assess illness severity at admission).

Variables with less than 20% missing values were imputed using the k-nearest neighbors (KNN) algorithm. Data preprocessing included standardization and conversion of categorical text data to numerical values to ensure dataset quality and accuracy. Continuous variables were retained in their original form, while binary variables were encoded. Lasso regression was subsequently applied for feature selection to identify core predictive variables.

### 2.3 Feature selection

Lasso regression analysis was employed for feature selection and high-dimensional data modeling. This method applies an L1 regularization penalty to regression coefficients, shrinking some coefficients to zero, thereby achieving feature selection and model simplification. Using sepsis as the dependent variable, a 10-fold cross-validation analysis was performed to determine the optimal regularization parameter. Based on the lambda.1se threshold (lambda value within one standard error of the optimal), 11 variables with non-zero coefficients were retained as significant predictors of sepsis risk.

### 2.4 Machine learning model construction and validation

After feature selection, the data were randomly split into a training set (*n* = 841) and a test set (*n* = 211) in an 8:2 ratio using the *createDataPartition* function from the R caret package. Set.seed(12345) was used to ensure reproducible grouping. We constructed 14 machine learning models, categorized as follows: Linear models: Logistic Regression, Lasso, Partial Least Squares, Discriminant Analysis; Tree-based and ensemble models: Random Forest, Gradient Boosting, XGBoost, CATBoost, LightGBM, Boosting Method; Other models: Kernel-based SVM, K-Nearest Neighbors, Neural Network, Naive Bayes.

We used 5-fold cross-validation to optimize model hyperparameters on the training set and evaluated model performance on the test set. Key evaluation metrics included sensitivity, specificity, accuracy, F1 score, Youden index, and AUC. Model performance was further assessed using receiver operating characteristic (ROC) curves, residual distribution plots, calibration curves, and DCA. SHAP analysis was used to interpret the prediction mechanisms of the ML models and quantify the contribution of each feature.

### 2.5 Model interpretation and feature importance

The SHAP method, grounded in game theory, was employed to interpret the output of the machine learning models (6). By computing SHAP values, this method quantifies the contribution of each predictor to the model’s outcome for individual predictions. Individual contributions were visualized using the R package shapviz (v.0.9.6). Notably, the SHAP method integrates both local and global interpretability. We applied this technique to establish an interpretable framework for the sepsis risk prediction model.

### 2.6 Simplification of the best machine learning prediction model

This study aimed to develop a streamlined model with optimal predictive performance by leveraging SHAP-based feature importance rankings. This simplified model reduces the complexity of clinical decision-making, allowing clinicians to quickly assess patient risk in routine practice and thereby improving the efficiency and accuracy of clinical decisions.

### 2.7 Statistical analysis

All statistical analyses were performed using SPSS 26.0 software (IBM Corporation, Armonk, New York, USA) and R 4.4.2 software (R Foundation for Statistical Computing, Vienna, Austria). RStudio was used for data processing, analysis, and machine learning model construction. Continuous variables with a non-normal distribution were presented as median and interquartile range (IQR), while normally distributed data were expressed as mean ± standard deviation (SD). Categorical variables were presented as frequencies and percentages (%), and group differences were compared using the Pearson chi-square test. A *p*-value <0.05 was considered statistically significant.

## 3 Results

### 3.1 Baseline characteristics of the study subjects

A total of 1,052 eligible patients with non-traumatic SAH were enrolled from the MIMIC-IV database and randomly divided into a training set (*n* = 841) and a test set (*n* = 211) in an 8:2 ratio. Baseline characteristics were well-balanced between the two groups (Table 1). This study initially included 44 potential predictive variables. Lasso regression was used for feature selection, and statistical tests were performed between the sepsis and non-sepsis groups. Results showed statistically significant between-group differences for all variables (all *p* < 0.001) (Table 2).

**Table 1:** Comparison of baseline data between training set and test set.

Variables	Total (*n* = 1,052)	Test (*n* = 211)	Train (*n* = 841)	*p*
Age, M (Q_1_, Q_3_)	61.00 (51.00, 72.00)	59.00 (51.00, 71.00)	62.00 (51.00, 73.00)	0.163
Charlson comorbidity index, M (Q_1_, Q_3_)	4.00 (2.00, 6.00)	3.00 (2.00, 5.00)	4.00 (2.00, 6.00)	0.219
HR, M (Q_1_, Q_3_)	80.00 (70.00, 91.00)	80.00 (69.50, 90.00)	80.00 (70.00, 91.00)	0.955
SBP, M (Q_1_, Q_3_)	128.98 (114.00, 142.00)	125.00 (114.00, 141.00)	129.00 (115.00, 143.00)	0.169
DBP, M (Q_1_, Q_3_)	70.00 (62.00, 80.00)	70.00 (63.00, 81.00)	70.00 (61.00, 80.00)	0.321
MAP, M (Q_1_, Q_3_)	86.00 (76.00, 95.00)	85.00 (77.50, 95.00)	86.00 (76.00, 95.00)	0.903
RR, M (Q_1_, Q_3_)	17.00 (15.00, 20.00)	17.00 (15.00, 20.00)	17.00 (15.00, 21.00)	0.618
SPO2, M (Q_1_, Q_3_)	98.00 (96.00, 100.00)	99.00 (96.00, 100.00)	98.00 (96.00, 100.00)	0.412
Initial temperature, M (Q_1_, Q_3_)	36.83 (36.56, 37.11)	36.89 (36.56, 37.17)	36.83 (36.56, 37.11)	0.501
WBC, M (Q_1_, Q_3_)	11.20 (8.60, 14.40)	11.40 (8.75, 14.95)	11.20 (8.50, 14.20)	0.215
RBC, M (Q_1_, Q_3_)	4.04 (3.66, 4.41)	4.07 (3.60, 4.45)	4.02 (3.66, 4.40)	0.807
Platelets, M (Q_1_, Q_3_)	214.50 (173.00, 261.00)	219.00 (175.50, 267.50)	213.00 (172.00, 256.00)	0.350
Hemoglobin, M (Q_1_, Q_3_)	12.20 (11.00, 13.40)	12.30 (11.15, 13.50)	12.20 (11.00, 13.40)	0.595
RDW, M (Q_1_, Q_3_)	13.40 (12.80, 14.30)	13.40 (12.85, 14.05)	13.40 (12.80, 14.30)	0.601
Hematocrit, M (Q_1_, Q_3_)	36.70 (33.18, 39.80)	37.10 (33.10, 40.10)	36.60 (33.20, 39.60)	0.555
Sodium, M (Q_1_, Q_3_)	139.00 (137.00, 141.00)	139.00 (137.00, 141.00)	139.00 (137.00, 141.00)	0.585
Potassium, M (Q_1_, Q_3_)	3.90 (3.60, 4.20)	3.90 (3.60, 4.30)	3.90 (3.60, 4.20)	0.560
Magnesium, M (Q_1_, Q_3_)	1.90 (1.70, 2.02)	1.90 (1.70, 2.00)	1.90 (1.70, 2.10)	0.356
Calcium total, M (Q_1_, Q_3_)	8.60 (8.20, 9.00)	8.60 (8.20, 9.00)	8.60 (8.20, 9.00)	0.796
Chloride, M (Q_1_, Q_3_)	105.00 (102.00, 108.00)	105.00 (102.00, 108.00)	105.00 (102.00, 108.00)	0.954
Glucose, M (Q_1_, Q_3_)	130.00 (110.00, 156.45)	131.00 (111.00, 158.00)	129.00 (110.00, 156.00)	0.729
Anion gap, M (Q_1_, Q_3_)	14.00 (12.00, 16.00)	14.00 (12.00, 16.00)	14.00 (12.00, 16.00)	0.797
Bicarbonate, M (Q_1_, Q_3_)	23.00 (21.00, 25.00)	22.01 (21.00, 24.00)	23.00 (21.00, 25.00)	0.624
PT, M (Q_1_, Q_3_)	12.40 (11.62, 13.40)	12.30 (11.60, 13.20)	12.40 (11.70, 13.40)	0.518
APTT, M (Q_1_, Q_3_)	28.10 (25.80, 31.10)	27.90 (25.95, 31.40)	28.10 (25.70, 31.00)	0.786
INR, M (Q_1_, Q_3_)	1.10 (1.10, 1.20)	1.10 (1.10, 1.20)	1.10 (1.10, 1.20)	0.769
Urea nitrogen, M (Q_1_, Q_3_)	13.00 (10.00, 18.00)	13.00 (10.00, 17.00)	13.00 (10.00, 18.00)	0.140
Creatinine, M (Q_1_, Q_3_)	0.80 (0.60, 1.00)	0.80 (0.60, 0.90)	0.80 (0.60, 1.00)	0.209
Sepsis, *n* (%)	505 (48.00)	101 (47.87)	404 (48.04)	0.965
AKI, *n* (%)	782 (74.33)	150 (71.09)	632 (75.15)	0.228
Pneumonia, *n* (%)	224 (21.29)	45 (21.33)	179 (21.28)	0.989
Gender, *n* (%)				0.075
Men	451 (42.87)	79 (37.44)	372 (44.23)	
Women	601 (57.13)	132 (62.56)	469 (55.77)	
Race, *n* (%)				0.229
White	613 (58.27)	124 (58.77)	489 (58.15)	
Black	38 (3.61)	11 (5.21)	27 (3.21)	
Asian	75 (7.13)	20 (9.48)	55 (6.54)	
Other	213 (20.25)	37 (17.54)	176 (20.93)	
Unknown	113 (10.74)	19 (9.00)	94 (11.18)	
GCS grade, *n* (%)				0.311
13–15	784 (74.52)	165 (78.20)	619 (73.60)	
9–12	128 (12.17)	19 (9.00)	109 (12.96)	
6–8	82 (7.79)	18 (8.53)	64 (7.61)	
3–5	58 (5.51)	9 (4.27)	49 (5.83)	
Hypertension, *n* (%)	545 (51.81)	113 (53.55)	432 (51.37)	0.570
Diabetes, *n* (%)	162 (15.40)	33 (15.64)	129 (15.34)	0.914
Myocardial infarction, *n* (%)	35 (3.33)	7 (3.32)	28 (3.33)	0.993
Malignant tumor, *n* (%)	96 (9.13)	15 (7.11)	81 (9.63)	0.255
Chronic kidney disease, *n* (%)	64 (6.08)	9 (4.27)	55 (6.54)	0.217
Mechanical ventilation, *n* (%)	794 (75.48)	151 (71.56)	643 (76.46)	0.140
Dopamine, *n* (%)	13 (1.24)	3 (1.42)	10 (1.19)	1.000
Epinephrine, *n* (%)	26 (2.47)	5 (2.37)	21 (2.50)	0.915
Norepinephrine, *n* (%)	189 (17.97)	29 (13.74)	160 (19.02)	0.074

Between-group comparison of baseline characteristics showed *p* > 0.05, indicating good balance for model training and validation.

ICU, intensive care unit; WBC, white blood cell; RBC, red blood cell; RDW, red cell distribution width; PT, prothrombin time; APTT, activated partial thromboplastin time; INR, International Normalized Ratio; HR, heart rate; SBP, systolic blood pressure; DBP, diastolic blood pressure; MAP, mean arterial pressure; RR, respiratory rate; SpO_2_, percutaneous oxygen saturation; AG, anion gap; GCS, Glasgow Coma Score; AKI, acute kidney injury. Data are presented as median (IQR) or *n* (%). Statistical tests: Kruskal–Wallis rank sum test for continuous variables; Pearson’s chi-squared test for categorical variables.

**Table 2:** Comparison of variables between sepsis and non-sepsis groups.

Variables	Total (*n* = 1,052)	Non-sepsis (*n* = 547)	Sepsis (*n* = 505)	*p*
RR, M (Q_1_, Q_3_)	17.00 (15.00, 20.00)	17.00 (15.00, 19.00)	18.00 (15.00, 21.00)	<0.001
WBC, M (Q_1_, Q_3_)	11.20 (8.60, 14.40)	10.30 (8.00, 12.90)	12.30 (9.60, 15.80)	<0.001
RBC, M (Q_1_, Q_3_)	4.04 (3.66, 4.41)	4.10 (3.72, 4.45)	3.96 (3.53, 4.37)	<0.001
RDW, M (Q_1_, Q_3_)	13.40 (12.80, 14.30)	13.20 (12.70, 13.80)	13.60 (13.00, 14.60)	<0.001
Glucose, M (Q_1_, Q_3_)	130.00 (110.00, 156.45)	121.00 (106.00, 143.50)	138.00 (118.00, 175.00)	<0.001
AKI, *n* (%)	782 (74.33)	349 (63.80)	433 (85.74)	<0.001
Pneumonia, *n* (%)	224 (21.29)	20 (3.66)	204 (40.40)	<0.001
Race, *n* (%)				<0.001
White	613 (58.27)	357 (65.27)	256 (50.69)	
Black	38 (3.61)	18 (3.29)	20 (3.96)	
Asian	75 (7.13)	42 (7.68)	33 (6.53)	
Other	213 (20.25)	77 (14.08)	136 (26.93)	
Unknown	113 (10.74)	53 (9.69)	60 (11.88)	
GCS grade, *n* (%)				<0.001
13–15	784 (74.52)	461 (84.28)	323 (63.96)	
9–12	128 (12.17)	54 (9.87)	74 (14.65)	
6–8	82 (7.79)	19 (3.47)	63 (12.48)	
3–5	58 (5.51)	13 (2.38)	45 (8.91)	
Mechanical ventilation, *n* (%)	794 (75.48)	331 (60.51)	463 (91.68)	<0.001
Norepinephrine, *n* (%)	189 (17.97)	27 (4.94)	162 (32.08)	<0.001

RR, respiratory rate; WBC, white blood cell; RBC, red blood cell; RDW, red cell distribution width; AKI, acute kidney injury. Data are presented as median (IQR) or *n* (%). Statistical tests: *Z*-test for continuous variables; *χ*^2^ test for categorical variables.

### 3.2 Feature selection results

**Figure 2:**
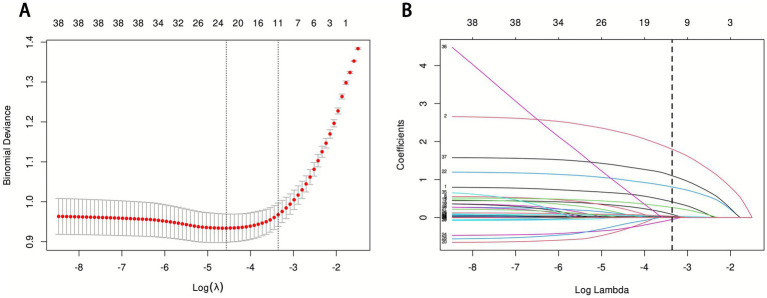
LASSO regression analysis results. **(A)** LASSO regression model feature selection: The left dashed line represents the optimal lambda value (lambda·min), and the right dashed line marks the lambda value within one standard error of the optimal (lambda.1se); **(B)** Trajectory of variables screened by the LASSO regression model.

This study initially included 44 potential influencing factors, covering demographic characteristics, comorbidities, underlying diseases, physiological indicators, scores, and laboratory parameters. After Lasso regression screening (*λ* = 0.0348480891087502), 11 potential predictors were retained (Figure 2). Figure 2A shows the lambda selection via 10-fold cross-validation (*λ* = 0.0348480891087502) to balance complexity and performance; Figure 2B illustrates variable coefficient trajectories, with 11 features retaining non-zero coefficients after compression. The final selected features were: AKI, pneumonia, race, GCS grade, RR, mechanical ventilation, WBC, RBC, RDW, glucose, and norepinephrine use.

### 3.3 Model performance evaluation results

**Figure 3:**
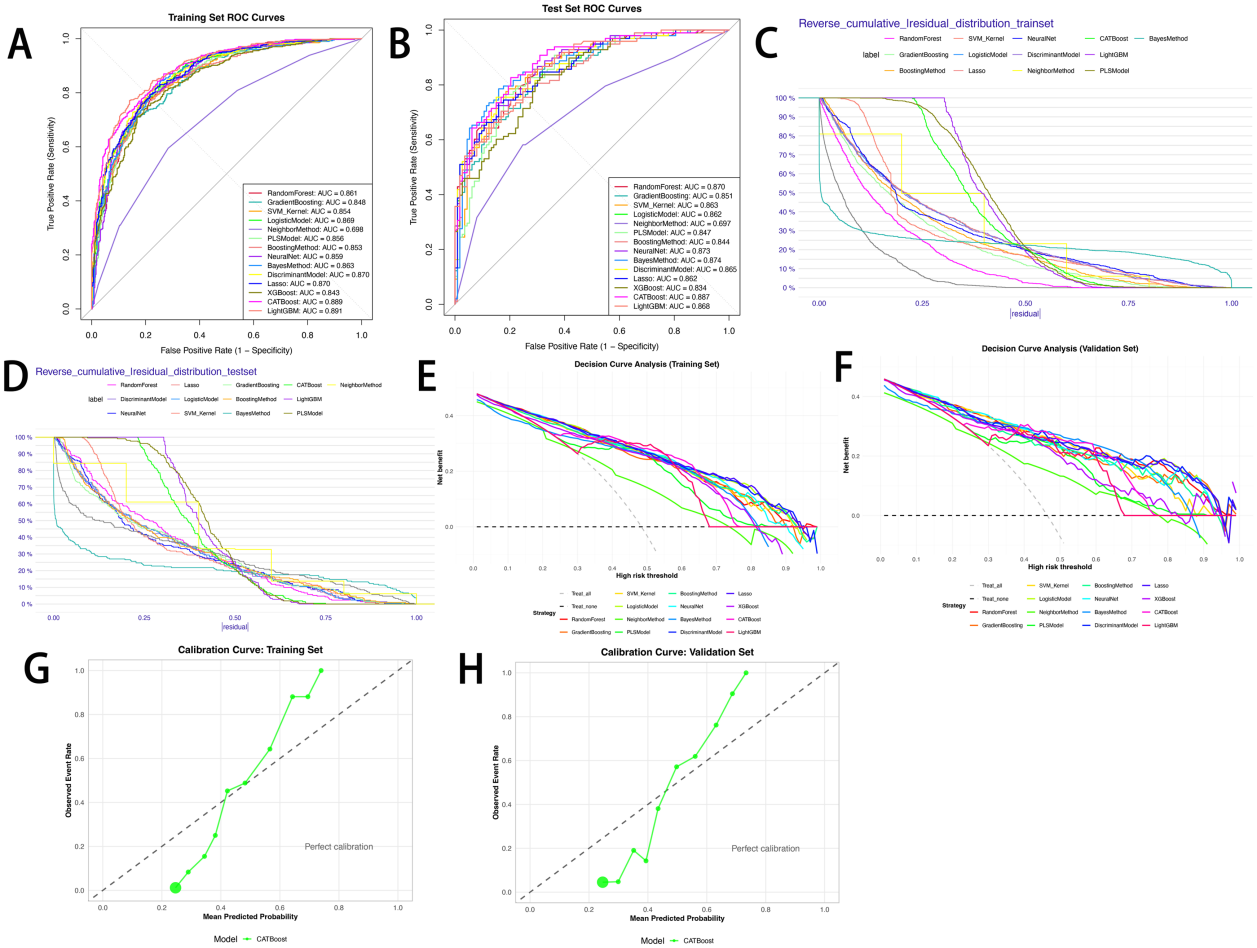
Performance comparison of 14 predictive models. **(A)** ROC curves for the training set; **(B)** ROC curves for the test set; **(C)** reverse cumulative residual distribution for the training set; **(D)** reverse cumulative residual distribution for the test set; **(E)** decision curve analysis (DCA) for the training set; **(F)** decision curve analysis (DCA) for the test set; **(G)** calibration curve for the CATBoost model (training set); **(H)** calibration curve for the CATBoost model (test set).

Fourteen machine learning models were constructed to predict the risk of sepsis (Figure 3). Model performance was evaluated using ROC curves, residual distribution plots, calibration curves, and DCA. By integrating the 11 core predictors, the CATBoost model exhibited the most robust and effective predictive performance in both the training and test sets.

The CATBoost model demonstrated excellent discriminative ability: in the training set, it achieved an AUC of 0.889 (95% CI: 0.868–0.911), accuracy of 79.8%, sensitivity of 73.2%, precision of 83.0%, specificity of 85.9%, F1 score of 77.8%, and a Youden index of 0.592 (Figure 3A). In the test set, it achieved an AUC of 0.887 (95% CI: 0.844–0.931), accuracy of 79.1%, sensitivity of 75.5%, precision of 78.7%, specificity of 82.3%, F1 score of 77.1%, and a Youden index of 0.578 (Figure 3B). Notably, the difference in AUC between the training and test sets was only 0.002, with performance fluctuation less than 2%, highlighting the model’s excellent stability. Among the 14 models, the CATBoost model achieved the highest Youden index (0.592 in training, 0.578 in test) and balanced sensitivity/specificity, outperforming XGBoost (Youden index: 0.542 in training, 0.472 in test) and LightGBM (0.613 in training, 0.487 in test) (Tables 3, 4).

**Table 3:** Performance metrics of machine learning models on the training set.

Models	Sensitivity	Specificity	Accuracy	PPV	NPV	F1 score	Youden’s index	AUC 95% CI
Random forest	0.747	0.793	0.771	0.772	0.770	0.759	0.540	0.861 (0.837–0.886)
Gradient boosting	0.740	0.776	0.759	0.756	0.761	0.748	0.516	0.848 (0.823–0.874)
SVM_Kernel	0.776	0.790	0.784	0.776	0.790	0.776	0.567	0.854 (0.829–0.879)
Logistic model	0.730	0.827	0.780	0.798	0.765	0.763	0.557	0.869 (0.845–0.892)
Neighbor method	0.595	0.717	0.658	0.663	0.653	0.627	0.311	0.698 (0.664–0.733)
PLS model	0.722	0.829	0.778	0.799	0.761	0.759	0.552	0.856 (0.831–0.881)
Boosting method	0.759	0.806	0.784	0.786	0.781	0.773	0.566	0.853 (0.828–0.878)
Neural net	0.786	0.786	0.786	0.775	0.797	0.780	0.572	0.859 (0.834–0.884)
Bayes method	0.713	0.832	0.774	0.799	0.755	0.753	0.544	0.863 (0.838–0.887)
Discriminant model	0.715	0.848	0.784	0.815	0.760	0.762	0.563	0.870 (0.847–0.894)
Lasso	0.727	0.832	0.781	0.802	0.765	0.763	0.559	0.870 (0.847–0.894)
XGBoost	0.752	0.790	0.772	0.771	0.773	0.761	0.542	0.843 (0.817–0.869)
CATBoost	0.732	0.859	0.798	0.830	0.774	0.778	0.592	0.889 (0.868–0.911)
LightGBM	0.786	0.827	0.807	0.810	0.805	0.798	0.613	0.891 (0.870–0.913)

PPV, positive predictive value; NPV, negative predictive value; AUC, area under the receiver operating characteristic curve; CI, confidence interval.

**Table 4:** Performance metrics of machine learning models on the test set.

Models	Sensitivity	Specificity	Accuracy	PPV	NPV	F1 score	Youden’s index	AUC 95% CI
Random forest	0.745	0.796	0.773	0.760	0.783	0.753	0.541	0.870 (0.823–0.917)
Gradient boosting	0.735	0.761	0.749	0.727	0.768	0.731	0.496	0.851 (0.801–0.901)
SVM_Kernel	0.776	0.814	0.796	0.784	0.807	0.779	0.590	0.863 (0.814–0.912)
Logistic model	0.714	0.841	0.782	0.795	0.772	0.753	0.555	0.862 (0.814–0.910)
Neighbor method	0.582	0.743	0.668	0.663	0.672	0.620	0.325	0.697 (0.627–0.768)
PLS model	0.704	0.823	0.768	0.775	0.762	0.738	0.527	0.847 (0.795–0.898)
Boosting method	0.735	0.796	0.768	0.758	0.776	0.746	0.531	0.844 (0.792–0.896)
Neural net	0.786	0.743	0.763	0.726	0.800	0.755	0.529	0.873 (0.828–0.919)
Bayes method	0.714	0.885	0.806	0.843	0.781	0.773	0.599	0.874 (0.826–0.922)
Discriminant model	0.694	0.858	0.782	0.810	0.764	0.747	0.552	0.865 (0.817–0.913)
Lasso	0.714	0.841	0.782	0.795	0.772	0.753	0.555	0.862 (0.814–0.911)
XGBoost	0.755	0.717	0.735	0.698	0.771	0.725	0.472	0.834 (0.781–0.887)
CATBoost	0.755	0.823	0.791	0.787	0.795	0.771	0.578	0.887 (0.844–0.931)
LightGBM	0.735	0.752	0.744	0.720	0.766	0.727	0.487	0.868 (0.821–0.915)

PPV, positive predictive value; NPV, negative predictive value; AUC, area under the receiver operating characteristic curve; CI, confidence interval.

In residual analysis, the CATBoost model showed optimal error control. In the training set, the median absolute residual was 0.20 (IQR = 0.10, RMSE = 0.22); when |residual| ≤ 0.25, the cumulative proportion of low-error samples reached 75%–80% (Figure 3C). In the test set, the median absolute residual was 0.21 (IQR = 0.11, RMSE = 0.23), and the cumulative proportion for |residual| ≤ 0.25 remained at 70%–75% (Figure 3D). No significant error degradation was observed after generalization, confirming reliable predictive ability for unseen data.

Calibration curve results showed that the CATBoost model’s actual event rate curves closely followed the “perfect calibration line” in both the training set (Hosmer–Lemeshow test: *χ*^2^ = 28.45, *p* = 0.15; Brier score = 0.142) and the test set (Hosmer–Lemeshow test: *χ*^2^ = 32.17, *p* = 0.09; Brier score = 0.156), indicating that predicted probabilities accurately translated to actual sepsis risks.

DCA further validated the clinical utility of the CATBoost model. In the training set (Figure 3E), all 14 models achieved positive net benefits within the risk threshold range of 0.0–1.0, significantly outperforming the “treat all” and “treat none” strategies. The CATBoost model achieved the highest net benefit within the clinically relevant threshold range of 0.2–0.6, reaching a net benefit of 0.45–0.50 at a threshold of 0.3. This was 0.10–0.15 higher than the “treat all” strategy and 0.30–0.35 higher than the “treat none” strategy, effectively reducing both “over-intervention” in low-risk patients and “missed diagnosis” in high-risk patients. In the test set (Figure 3F), the CATBoost model maintained a net benefit of 0.40–0.45 in the threshold range of 0.2–0.6, only slightly lower than in the training set. In contrast, models such as LightGBM and XGBoost showed more significant declines in net benefit (a decrease of 0.10–0.12). Specifically, in the threshold range of 0.3–0.5, the CATBoost model’s net benefit was 0.05–0.08 higher than that of LightGBM, confirming its stable utility in clinical decision-making. Additionally, the calibration curves confirmed the stability of the results for CATBoost in training set (Figure 3G) and test set (Figure 3H). Additionally, Figures 3C,D display the CATBoost model’s concentrated residual distribution (median absolute residual: 0.20 in training, 0.21 in test), indicating minimal prediction error; Figures 3G,H confirm good calibration (Hosmer-Lemeshow test *p* > 0.05), ensuring predicted probabilities align with actual sepsis risks.

Compared with other models, LightGBM achieved the highest AUC in the training set (0.891), while Naive Bayes had the second-highest AUC in the test set (0.874). However, LightGBM showed signs of overfitting: its test set AUC dropped to 0.868, and specificity decreased from 0.827 to 0.752. Although Gradient Boosting, Kernel-based SVM, Logistic Regression, and Neural Network also demonstrated strong predictive performance (training set AUC: 0.848–0.870; test set AUC: 0.834–0.874), the CATBoost model performed more consistently across datasets, particularly in sensitivity, specificity, and Youden index on the test set. Furthermore, it exhibited a more concentrated residual distribution and higher net clinical benefit, leading to its selection as the optimal model.

### 3.4 Model visualization based on SHAP principle

**Figure 4:**
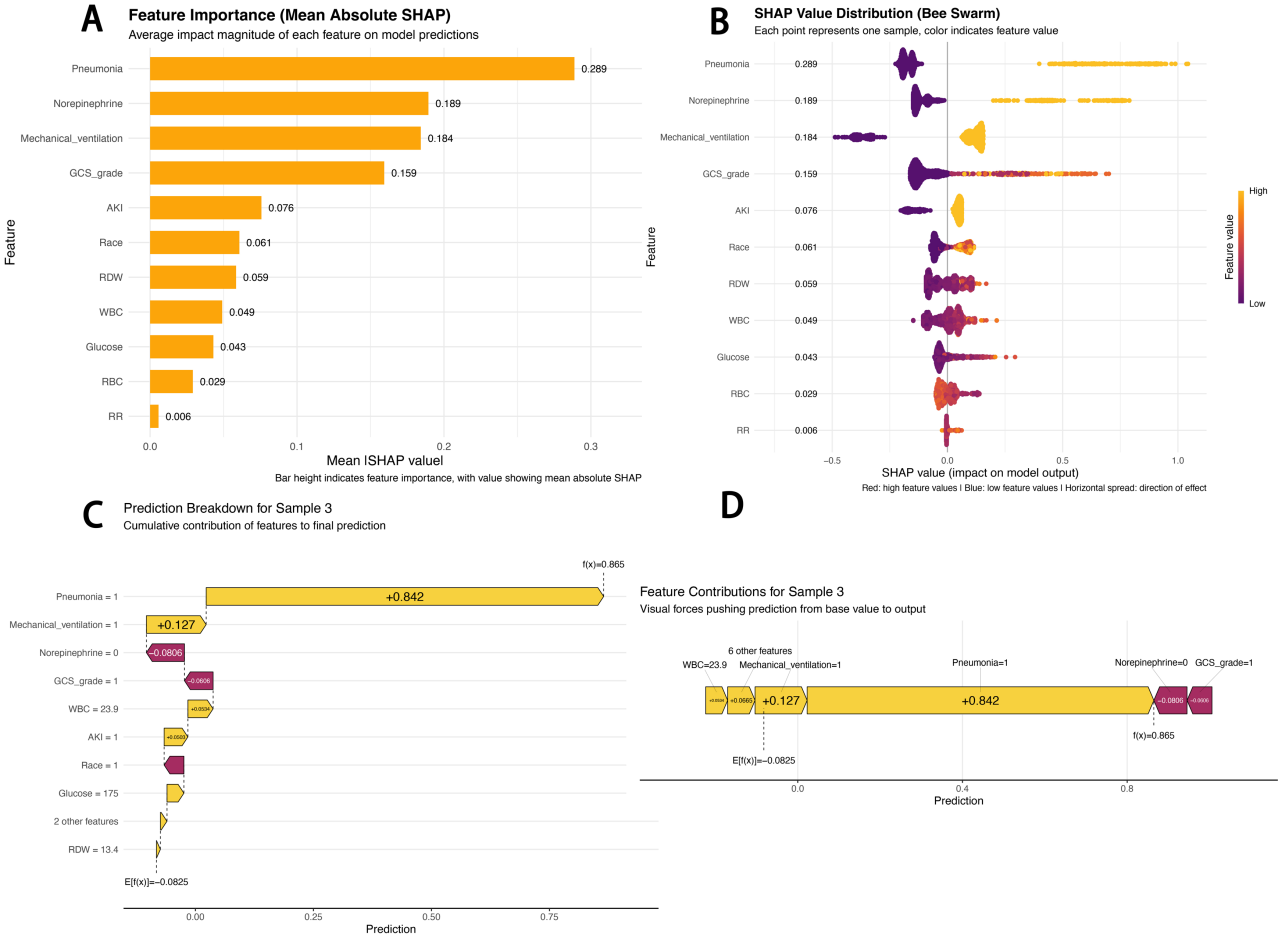
SHAP-based model interpretation. **(A)** SHAP feature importance (mean absolute SHAP value): Bar height indicates feature importance, with numerical values representing mean absolute SHAP values; **(B)** SHAP beeswarm plot: Red dots indicate high feature values, blue dots indicate low feature values, and the horizontal spread reflects the direction of the feature’s impact; **(C)** SHAP waterfall chart for sample 3: Decomposes the cumulative contribution of each feature to the final prediction; **(D)** SHAP force-directed diagram for sample 3: Visualizes the “forces” pushing the prediction from the baseline value to the final output.

The SHAP feature importance bar chart shows that pneumonia (mean absolute SHAP value: 0.289) was the most important predictive feature, followed by norepinephrine use (0.189) and mechanical ventilation (0.184), while respiratory rate (0.006) had the minimal impact (Figure 4A).

The SHAP beeswarm plot reveals the predictive mechanism of the CATBoost model (Figure 4B). The horizontal axis represents the SHAP value (impact on model output), and the vertical axis displays features ranked by their cumulative SHAP value influence. Each point corresponds to a specific patient sample. For pneumonia, samples with pneumonia mostly had positive SHAP values (0.4–0.8), indicating an increased predicted sepsis risk; in contrast, samples without pneumonia mostly had negative SHAP values (−0.2 to 0), indicating a reduced risk. Red dots for norepinephrine use and mechanical ventilation were also primarily distributed in the positive SHAP value range, confirming that these factors significantly increase sepsis risk.

The SHAP waterfall chart and force-directed diagram (Figures 4C,D) illustrate the prediction logic for a typical high-risk patient (Sample 3). The model predicted a high sepsis risk [*f*(*x*) = 0.865] based on the following logic: starting from the baseline risk (*E*[*f*(*x*)] = −0.0825), pneumonia (Pneumonia = 1) was the strongest positive contributor (+0.842), and mechanical ventilation (Mechanical_ventilation = 1, +0.127) further elevated the risk. Although the absence of norepinephrine use (−0.0806) and a high GCS score (GCS = 1, indicating 13–15 points, −0.0606) slightly reduced the risk, elevated white blood cell count (WBC = 23.9, +0.0534) and acute kidney injury (AKI = 1, +0.0503) ultimately contributed to the high-risk prediction. These visualizations intuitively present the contribution of each feature to individual risk predictions, providing an interpretable basis for the model’s decision-making logic.

## 4 Discussion

This study developed and validated a machine learning model using the MIMIC-IV database to predict sepsis risk in patients with non-traumatic SAH. Lasso regression identified 11 predictive variables: AKI, pneumonia, race, GCS grade, RR, mechanical ventilation, WBC, RBC, RDW, glucose, and norepinephrine use. The CATBoost model exhibited excellent discriminative performance in both the training and test sets. Compared with the SAH-specific sepsis nomogram (5) and general ICU sepsis prediction models (7), our CATBoost model achieves higher predictive accuracy (AUC = 0.887) with interpretability via SHAP analysis, addressing unmet clinical needs for this vulnerable subgroup.

Systemic Inflammatory Response Syndrome (SIRS) is common after SAH and shares many clinical features with sepsis, making timely diagnosis and intervention challenging in this patient population (3). Recent advances in machine learning have revolutionized predictive modeling in critical care, providing powerful tools for risk stratification and prognosis prediction (8). These computational methods can process complex, high-dimensional data and identify subtle patterns that are difficult to detect with traditional statistical approaches (9). Several studies have successfully applied machine learning algorithms to predict various clinical outcomes, such as severe influenza in children (10), retinopathy of prematurity (11), causes of liver abscesses (12), depression risk (13), and delayed medical treatment in cancer patients (14). The flexibility of machine learning models allows for the inclusion of numerous variables, facilitating the development of robust prediction tools (15).

The performance of the CATBoost model must also be interpreted in the practical context of ICU operations. Its high specificity (82.3%–85.9%) is clinically crucial: in resource-limited ICU settings, a model with low specificity would generate excessive false alarms, leading to alert fatigue and potential misuse of broad-spectrum antibiotics (7). By reliably identifying patients at truly low risk, the model helps clinicians focus vigilance and resources on high-risk individuals, supporting antimicrobial stewardship efforts. This is particularly relevant for SAH patients, as initial SIRS can mimic sepsis, making early and accurate discrimination challenging.

Furthermore, the selected predictive features align with the known pathophysiological trajectory of severe non-traumatic SAH, effectively capturing the cascade of events from initial neurological insult to heightened systemic infection risk. For example, a low GCS score predisposes patients to aspiration, while mechanical ventilation breaches innate airway defenses, collectively increasing the risk of pneumonia, which emerged as the top predictor in our model (16). Subsequent circulatory failure and organ dysfunction represent progression toward a sepsis phenotype. Thus, the model not only identifies statistical associations but also encapsulates a clinically recognizable sequence of neurological injury, complication development, and systemic deterioration.

Compared with traditional predictive methods, integrating inflammatory markers, clinical parameters, and laboratory values via advanced computational approaches achieves higher prediction accuracy (17). Additionally, feature importance analysis using methods like SHAP improves model interpretability, a common limitation of complex algorithms (13). In clinical practice, interpretability is critical: clarifying the relative contributions of predictors is essential for effective model application and clinical decision support (17).

In this study, the CATBoost model achieved an AUC exceeding 0.88 and a Youden index >0.57 in both the training and test sets, significantly outperforming traditional models and other ensemble algorithms. Its advantages stem from three key attributes: first, CATBoost efficiently handles categorical features through built-in encoding, reducing preprocessing steps and minimizing information loss, an ideal characteristic for the mixed data types in this study (18). Second, it mitigates overfitting via ordered boosting, maintaining stable performance even on small-sample test sets and resolving the common “generalization gap” observed in other tree-based models (19). Third, it is robust to outliers, preventing anomalous data points from skewing model performance (20).

SHAP analysis revealed that pneumonia, norepinephrine use, mechanical ventilation, and GCS grade had the greatest impact on model output, with high values or the presence of these factors closely associated with increased sepsis risk. Non-traumatic SAH patients often have impaired swallowing reflexes and are at high risk of aspiration due to depressed consciousness. Prolonged bed rest also leads to poor pulmonary drainage, further increasing pneumonia incidence (21). Pneumonia can trigger sepsis via an “inflammatory cytokine storm–organ dysfunction” pathway; studies indicate that pneumonia is a leading cause and common infection source in sepsis, with the two conditions often coexisting to worsen illness severity and increase mortality (22).

Norepinephrine is commonly used in patients with circulatory failure. While our model shows a positive association between norepinephrine use and sepsis risk, it is critical to clarify that this reflects a clinical association rather than causation. Norepinephrine itself does not induce sepsis; its predictive value stems from its role as a surrogate marker of a high-risk physiological state. Clinically, norepinephrine is a first-line vasopressor for septic shock or severe infection-related hypotension, and its use typically precedes or coincides with sepsis diagnosis—indicating that the patient’s infection has progressed to require circulatory support (23). Additionally, non-traumatic SAH patients are susceptible to cerebral hypoperfusion due to elevated intracranial pressure and vasospasm, often requiring vasoactive drugs. However, norepinephrine can suppress immune function, and the invasive vascular access required for its administration increases infection risk, further strengthening the association between its use and sepsis (24).

Non-traumatic SAH patients may require mechanical ventilation due to respiratory depression or aspiration pneumonia. Mechanical ventilation disrupts the airway barrier, increasing the risk of ventilator-associated pneumonia (VAP), which affects 10%–40% of ventilated patients (25). Antibiotic use in this context further increases the risk of drug-resistant bacterial infections, elevating sepsis risk. A lower GCS score correlates with higher sepsis risk, as more severe consciousness impairment compromises self-protective reflexes and increases infection susceptibility (26). Elevated RDW and AKI are also important risk factors, highlighting that sepsis development is a multifactorial process involving underlying patient conditions, complications, and organ damage.

A key strength of this study is the application of the SHAP framework, which moves beyond “black box” predictions to provide clinically interpretable and actionable insights. The ability to quantify and visualize each feature’s contribution to an individual patient’s predicted risk represents a significant advancement over traditional logistic regression models, which only provide odds ratios. This interpretability is critical for building clinician trust and facilitating potential integration into clinical workflows. This approach aligns with best practices, as demonstrated by its successful application in models predicting treatment response in glioma (27), breast cancer malignancy (28), and pathological complete response in esophageal squamous cell carcinoma (29). By identifying pneumonia as the primary driver of sepsis risk, the model directs clinical attention to modifiable factors, enabling preemptive interventions such as enhanced ventilator care bundles or early antibiotic stewardship for high-risk patients.

The significant net benefit of the CATBoost model, demonstrated by DCA across the clinically relevant threshold range of 0.2–0.6, strongly supports its potential clinical utility. DCA confirms that using the model for decision-making yields a superior net benefit compared to the strategies of “treating all patients” or “treating no patients” with prophylactic interventions, assuming rational consideration of the harms of false positives and false negatives. This step is critical for translating statistical accuracy into practical clinical value, as seen in previous validations of models predicting critical influenza infection in children (10) and surgical site infections after colon surgery (30). The defined risk threshold range also provides clinicians with clear guidance on when the model’s predictions are most likely to inform beneficial clinical actions.

While the model shows promise, its ultimate value depends on integration into clinical workflows. Future work should focus on developing a simplified risk scoring system derived from the model’s key features to enhance bedside usability. Additionally, prospective validation in diverse, multi-center cohorts is essential to confirm its generalizability and assess its impact on hard clinical outcomes. Incorporating dynamic data could further refine predictive accuracy and enable real-time risk updates.

This study has several limitations. First, it is a database retrospective study, potentially introducing regional bias. The model therefore requires validation in multicenter prospective cohorts. Second, despite its superior performance, the complexity of the CATBoost model may hinder routine clinical implementation, as it may require specialized computing resources and technical support. Third, the study focused on sepsis risk; the temporal dynamics of risk factors and their impact on long-term outcomes remain unclear. Future studies should include extended follow-up to capture the full spectrum of disease progression.

In summary, this study developed a machine learning prediction model that effectively identifies the risk of sepsis in ICU patients with non-traumatic SAH. The model outperformed traditional scoring systems and was rendered interpretable through SHAP analysis, highlighting its potential for clinical application. While acknowledging the limitations, we believe these findings provide valuable insights for the early warning of sepsis in this patient population and pave the way for future multicenter prospective validation and optimization of clinical intervention strategies to reduce sepsis incidence.

## 5 Conclusion

The CATBoost model, constructed using data from 1,052 non-traumatic subarachnoid hemorrhage (SAH) ICU patients in the MIMIC-IV database, effectively predicts the risk of sepsis, achieving an AUC of 0.887 (95% CI: 0.844–0.931) on the test set, with a sensitivity of 75.5% and specificity of 82.3%. SHAP analysis identified pneumonia, norepinephrine use, and mechanical ventilation as the top risk drivers, underscoring the model’s interpretability and clinical utility. This model serves as a valuable risk stratification tool to guide targeted early interventions, reduce overtreatment, and ultimately improve outcomes in this vulnerable patient subgroup.

## Data Availability

The datasets presented in this study can be found in online repositories. The names of the repository/repositories and accession number(s) can be found at: All data files are available from the MIMIC-IV database: https://physionet.org/content/mimiciv/3.0/.
